# Celiac Disease in Children with Idiopathic Nephrotic Syndrome—A Retrospective Cohort Study

**DOI:** 10.3390/jcm15010329

**Published:** 2026-01-01

**Authors:** Anna Ozimek, Wojciech Wasiak, Piotr Albrecht, Małgorzata Mizerska-Wasiak

**Affiliations:** 1 Department of Pediatrics and Nephrology, Medical University of Warsaw, 02-091 Warsaw, Poland; anna1ozimek@gmail.com; 2 Pediatrics Department, Warsaw Medical Care Center “KOPERNIK” Ltd., 00-328 Warsaw, Poland; wojtek.wasiak@gmail.com; 3 Department of Pediatric Gastroenterology and Nutrition, Medical University of Warsaw, 02-091 Warsaw, Poland

**Keywords:** idiopathic nephrotic syndrome, celiac disease, gluten-free diet, IgA deficiency, pediatric nephrology

## Abstract

**Objective**: Idiopathic nephrotic syndrome (INS) is a rare, relapsing kidney disease. Trigger for relapses, among others, may be exposure to gluten in patients with INS and celiac disease (CD). CD is a gluten-sensitive disorder. The prevalence of CD ranges from 1% in the general population to 8% in patients with autoimmune diseases. The aim of the study was to assess the incidence of CD in patients with INS and the influence of a gluten-free diet on the course of INS. **Material and Methods**: A retrospective cohort study was conducted on 147 patients hospitalized between February 2020 and September 2024 in a single medical center. Patients were categorized into two groups: 98 patients with INS and 49 from the control group. The analysis included age, gender, total dose of glucocorticoids (GCs), duration of INS, serum levels of immunoglobulin class A (IgA) and G (IgG), the presence of antibodies against tissue transglutaminase (tTG) and endomysium (EMA), and urine analysis. A medical questionnaire regarding pathological symptoms during infancy and allergic diseases of patients and family members was conducted. **Results**: CD was diagnosed in 8% of patients with INS. A total of 66% of patients with INS and CD who followed a gluten-free diet had no or less frequent relapses. **Conclusions**: CD is more common in patients with INS than in the general population. A gluten-free diet in patients with INS and CD may decrease the frequency of nephrotic proteinuria relapses. CD may be oligosymptomatic, and it is important to search for it in all patients with INS. Owing to the small number of patients with CD among INS in the study, this issue requires further research.

## 1. Introduction

Nephrotic syndrome (NS) is a group of symptoms defined by nephrotic proteinuria that leads to hypoalbuminemia, hyperlipidemia, and massive edemas. Nephrotic proteinuria is characterized by a urine protein-to-creatinine ratio above 2 mg/mg or urinary protein excretion above 50 mg/kg body weight per day in children or above 3.5 g per day in adults [1]. INS accounts for 90% of NS cases in children under 10 years of age and 50% in children older than 10 years of age [1]. The etiopathogenesis of INS is not fully understood and is likely to be multifactorial [2,3]. The triggers for recurrent nephrotic proteinuria may be infection, insect bites, contact with allergens for allergy sufferers or exposure to gluten in patients with CD [2,3,4]. In the literature, there are reports of attempts to implement a gluten-free diet in patients with INS that led to remission of proteinuria even without the use of GCs. In the studies, recurrence of proteinuria was observed after discontinuation of the gluten-free diet [4,5].

CD is a chronic, autoimmune, gluten-sensitive disorder that causes not only damage to the small intestinal mucosa but also dysfunction of multiple organs. It affects individuals with genetic predisposition (presence of HLA DQ2 and/or DQ8 histocompatibility antigens) [6]. Small-intestine mucosa damages after exposing to gluten are of various severity [6,7]. Gluten is a fraction of cereal proteins such as gliadin (wheat), hordein (barley), and secalin (rye). It is responsible for the flavor and baking quality of cereals. The amino acids of gluten proteins, primarily proline and glutamine, are pathogenic in the context of CD. Gluten fragments rich in proline and glutamine are deamidated by tissue transglutaminase to glutamic acid. These proteins are highly immunogenic and lead to mucosal damage in the small intestine [6,7]. Following the European Society for Pediatric Gastroenterology, Hepatology, and Nutrition (ESPGHAN) guidelines for diagnosis of CD in children, the diagnostic pathway should begin with an assessment of serum concentration of antibodies against tTG in IgA. If the concentration of antibodies exceeds 10 times the upper limit of normal (ULN) in two blood samples and serum antibodies against EMA are positive, there is no need for small-intestine mucosa biopsy. In case of IgA deficiency, tTG and EMA should be assessed in IgG [8,9]. Moreover, if the main symptom is skin lesions, called dermatitis herpetiformis or Duhring’s disease, the skin biopsy outcome is enough to confirm CD [10,11]. In other cases, histopathological assessment of a duodenal biopsy according to the modified MARH scale is needed [7,10]. This involves the presence of intraepithelial lymphocyte infiltration (grade 1), intestinal crypt hyperplasia (grade 2), and intestinal villus atrophy (grade 3a–c) [8,9].

Based on clinical and histopathological symptoms, there are four types of CD. Classic CD (gastrointestinal symptoms such as diarrhea, malabsorption, failure to thrive, and presence of tTG and/or EMA antibodies and/or MARSH ≥ 2), non-classic/atypical CD (silent or nonspecific symptoms like abdominal pain, constipation, presence of tTG, and/or EMA antibodies and/or MARSH ≥ 2), subclinical CD (no clinical symptoms, presence of tTG and/or EMA antibodies and/or MARSH ≥ 2), and potential CD (presence of tTG and/or EMA antibodies, no clinical symptoms, no histopathological changes) [8,9].

The aim of the study was to assess the prevalence of CD in patients with INS and its impact on the course of INS.

## 2. Materials and Methods

**Figure 1:**
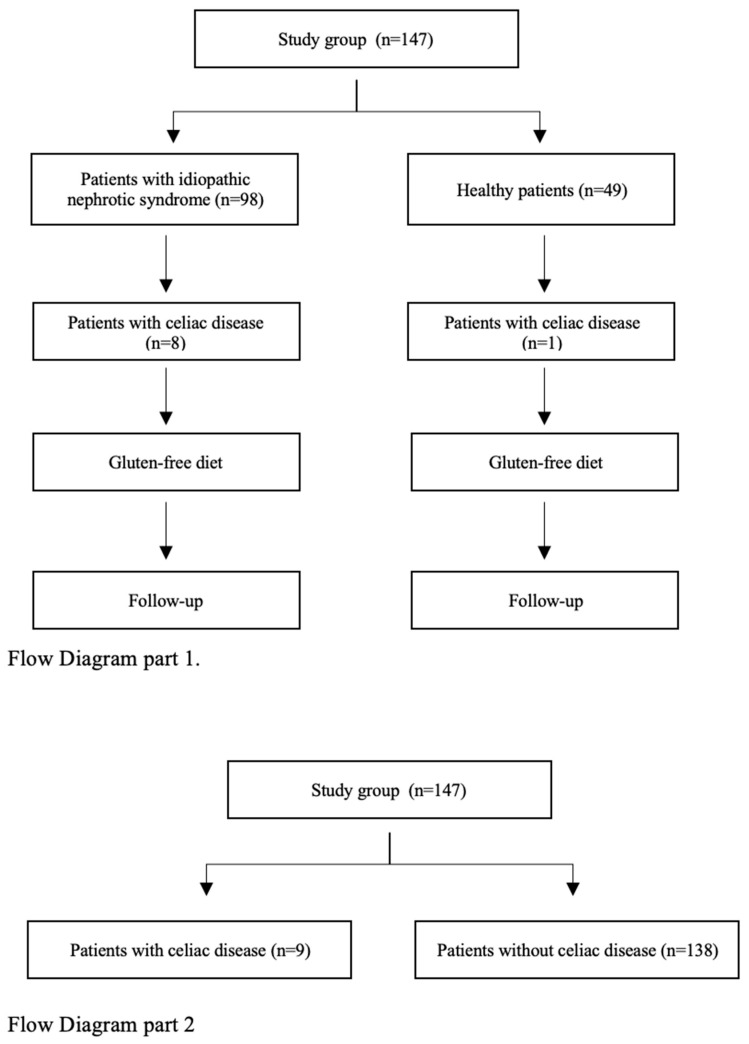
Flow diagram.

The study included 147 patients hospitalized at a single medical center between February 2020 and September 2024. The results of 98 patients with INS, which constitutes over 70% of the INS population of the single medical center, and 49 healthy patients or those with enuresis were assessed. Their medical history and medical tests outcomes were thoroughly studied. Inclusion criteria for the study group were age < 18 years old and a diagnosis of INS. Exclusion criteria were presence of nephropathy other than INS and a gluten-free diet. Before diagnosis of INS, antinuclear antibodies (ANAs), anti-neutrophic cytoplasmic antibodies (ANCA)s, abnormalities of C3 and C4 components of compliment, and infectious factors such as EBV (Ebstein–Barr virus), HBV (hepatitis B virus), HCV (hepatitis C virus), CMV (cytomegalovirus), and TBC (tuberculosis) were excluded. Inclusion criteria for the control group were age < 18 years old and no history of chronic diseases despite enuresis. Exclusion criteria were hypertension, glomerulonephritis, autoimmune disease, proteinuria, and a gluten-free diet. In all patients, age, gender, serum level of IgA and IgG, presence of antibodies against tTG and, in some cases, against EMA in IgA and IgG, and a urine analysis were assessed. In both groups, a medical questionnaire was conducted. It collected information about symptoms during infancy, such as prematurity, skin lesions, itching, abdominal pain, flatulence, infantile colic, constipation, diarrhea, mucus or blood in the stool, and failure to thrive. Furthermore, patients were asked about allergic rhinitis, bronchial asthma, allergic conjunctivitis, atopic dermatitis, rashes, CD, and gluten intolerance or allergy diagnoses (current or past) of patient and family members (parents, siblings, grandparents). In the study group (INS), the years of illness, total dose of GCs, current course of the disease (steroid sensitivity/resistance), and the number of relapses of nephrotic proteinuria (frequent/infrequent relapses) before and after the beginning of a gluten-free diet were assessed. The course of INS (frequently/infrequently recurring) and the effects of steroid therapy (steroid-sensitive/-resistant) were evaluated according to the Polish Society of Pediatric Nephrology (PTNfD) recommendations [1]. All patients with positive antibodies against tTG and/or EMA in IgA and/or IgG were consulted by a pediatric gastroenterologist. Further diagnostic workup for CD was based on the recommendations of ESPGHAN for diagnosis CD [9]. In a group of selected patients, gastroduodenoscopy with duodenal biopsy was performed. Histopathological evaluation of biopsy was based on the modified MARSH scale. After the diagnosis of CD, a gluten-free diet was recommended to patients in both groups. Patients diagnosed with potential CD remained under outpatient observation without dietary modifications. All CD patients were followed for at least 12 months after diagnosis. In the first part of the study, the impact of a gluten-free diet on the course of INS was assessed (Figure 1). In the second part of the study, medical questionnaire results were analyzed. Data from all patients diagnosed with CD (INS + healthy children) were compared with the group of children without CD (INS + healthy children) (Figure 1).

The study was conducted with the consent of the Medical University of Warsaw dated 16 November 2020, number KB/184/2020.

## 3. Statistics

The Lilliefors 2 test was used to verify the normality of the distribution of quantitative variables. Student’s *t*-test for independent samples was used to compare the mean differences in variables between two groups.

The Pearson chi-square test was used to compare the frequency of a given feature between groups. Additionally, the 95% confidence interval for the frequency of the feature in each group was determined.

Logistic regression was used to assess the correlation between the prevalence of celiac disease and qualitative features (gender, steroid exposure, frequent relapses) and quantitative features (age, disease duration).

The Statistica 13.PL statistical package and the R program with the PropCIs version 0-3.0 library were used for calculations.

## 4. Results

Children aged 1.52 to 17.79 years, with a median age of 8.12 years, including 82 (55%) boys and 67 (45%) girls, were enrolled in the study. There were 60 (60%) boys and 39 (40%) girls in the group of patients with INS and 22 (44.9%) boys and 27 (55.1%) girls in the control group. Within the INS group 89 (90%) patients had steroid-sensitive INS and 9 (9%) had steroid-resistant INS. The disease course was classified as frequently recurring in 48 (49%) and infrequently recurring in 50 (51%) patients. At the time of testing, GCs were used by 50 (51%) patients, including 7 of 8 (87.5%) patients with INS who were diagnosed with CD during the study.

**Figure 2:**
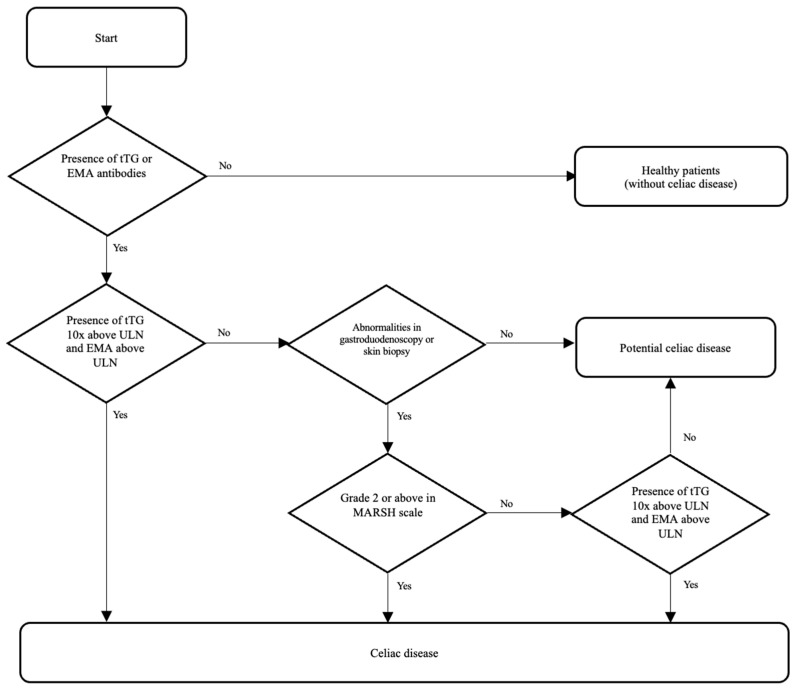
Flowchart of the diagnostics algorithm for celiac disease [tissue transglutaminase antibodies (tTG); endomysial antibodies (EMA); upper limit of normal (UNL); Modified Marsh classification (MARSH)].

**Figure 3:**
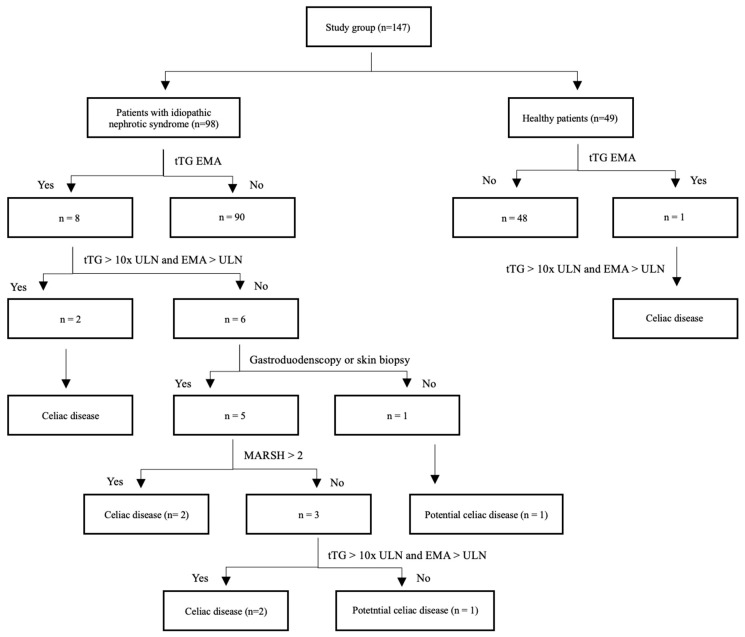
Number of patients presenting individual celiac disease markers in the INS group and in the control group [tissue transglutaminase antibodies (tTG); endomysial antibodies (EMA); upper limit of normal (UNL); Modified Marsh classification (MARSH)].

In the group of children with INS, positive titers of anti-tTG antibodies in IgA were detected in 8 of 98 (8%) patients and in IgG in 4 of 89 (4.5%) patients. Positive titers of anti-EMA antibodies in IgA were detected in 4 of 79 (5%) and in IgG in 1 of 78 (1.2%) patients. Based on IgA, anti-tTG antibodies titers 10× above ULN, and positive IgA and/or IgG EMA antibodies in the initial blood tests, CD was diagnosed in two of eight (25%) patients. Further diagnostics that included gastroduodenoscopy with duodenal biopsy were performed in four of eight (50%) and skin biopsy in one of eight (12.5%) patients with INS and suspicion of CD. Histopathological abnormalities in the duodenal biopsy specimen characteristic for CD (MARSH ≥ 2) were present in one of four (25%) patients. Another two patients, during follow-up, revealed increasing titers of IgA and/or IgG antibodies against tTG > 10× ULN and positive antibodies against EMA IgA and/or IgG. They were diagnosed with CD. Two more patients with normal gastroduodenoscopy results and/or anti-tTG antibody levels below 10× ULN were diagnosed with potential CD, kept a normal diet, and were followed-up in the outpatient clinic. The diagnostic path and study outcomes are presented in a flowchart (Figure 2), in Figure 3, and in Table 1.

Taking into account the course and GCs sensitivity of INS, CD was diagnosed in 1 of 9 (11%) patients with steroid-resistant INS, 7 of 89 (7.8%) with steroid-sensitive INS, 5 of 56 (8.9%) with infrequently recurring INS, and 1 of 42 (2.3%) with frequently recurring INS.

Moreover, IgA deficiency was diagnosed in 7 of 98 (7%) patients with INS. CD was diagnosed in three of seven (43%) patients with INS and IgA deficiency. In the control group, IgA deficiency was found in 4 of 49 (8%) patients. The one with CD had a normal IgA serum concentration. A genetic test for the HLA DQ2DQ8 allele was performed in one INS patient, and the presence of the DQ2 antigen was confirmed.

In the second part of the study, based on the medical questionnaire, patients with CD (eight patients with NS and one from the control group; n = 9) had a significantly higher incidence of skin rashes: prevalence of 0.7778 (95%Cl. 0.3999–0.9719) compared to the control group, at 0.3465 (95%Cl. 0.2643–0.4360); (*p* = 0.026); mucus in stools: prevalence of 0.444 (95%Cl, 0.1370–0.7880) compared to the control group, at 0.1094 (95%Cl. 0.0611–0.1767); (*p* = 0.018) in infancy; abdominal pain: prevalence of 0.5556 (95%Cl. 0.2120–0.8630) compared to the control group, at 0.2248 (95%Cl. 0.1560–0.3066); (*p* = 0.068); flatulence: prevalence of 0.5556 (95%Cl. 0.2120–0.8630) compared to the control group, at 0.2109 (95%Cl. 0.1438–0.2919) (*p* = 0.051); and failure to thrive: prevalence of 0.3750 (95%Cl. 0.0852–0.7551) compared to the control group, at 0.1094 (95%Cl. 0.0611–0.1767) (*p* = 0.098) in infancy were more common in patients with CD, with trends toward statistical significance. There were no significant differences in the susceptibility to CD in both groups regarding the week of pregnancy termination, skin pruritus, infantile colic, constipation, diarrhea, presence of blood in the stool in infancy, the frequency of atopic dermatitis, allergic rhinitis, bronchial asthma, skin lesions, allergic conjunctivitis in patients in the post-infancy period, and in family members (mother, father, grandparents, siblings) (Table 2).

By applying a multivariate stepwise regression test, it was validated that disease duration (*p* = 0.091) and steroid exposure (*p* = 0.126) were statistically correlated with CD with trends toward statistical significance. There were no significant differences in the susceptibility to CD regarding sex, age, and the course of INS (frequently/infrequently recurring).

Patients with INS and CD (n = 8) were followed for at least 12 months. Six of eight patients remained on a gluten-free diet, and two of eight with potential CD were on a gluten diet. Nephrotic proteinuria was not observed in three of six (50%) patients who had been on a gluten-free diet, and one of six (16%) experienced a reduction in the number of relapses. In another two patients, due to steroid resistance or high steroid dependence, the INS diagnostic workup was extended with renal biopsy and genetic tests. Moreover, second-line treatment (calcineurin inhibitors, levamisole) was initiated. In the history of patient 1, there was a renal biopsy that showed mesangial proliferation secondary to minimal change in disease, and a pathogenic PLCE1 variant was detected in one allele. Two years after initiating a gluten-free diet, the second-line treatment was discontinued, and nephroprotective therapy was maintained. Since then (3 years), the patient has experienced intermittent non-nephrotic proteinuria. In patient 2, a renal biopsy was performed that revealed mesangial proliferation, and no abnormalities in the genetic test were found. The gluten-free diet did not affect the course of the disease (Table 3).

## 5. Discussion

Our study found that the prevalence of CD in INS patients was 8% compared to 2% in the control group. According to the literature, serological features of CD are present in 1% of the general population and in 0.3 to 1.3% in the pediatric one [8,10]. Studies of the Polish pediatric population show that the prevalence of CD in school-age children is 0.4% (1:215) [10]. It is known that the prevalence of CD in patients with autoimmune diseases (type 1 diabetes, thyroiditis, juvenile idiopathic arthritis, myocarditis) is significantly higher and ranges from 1 to 8% [6,11]. The prevalence of CD in children with INS is similar to other high-risk populations. It may be purposeful to test INS patients for CD.

In the study, potential CD was initially diagnosed in four of eight (50%) patients. During follow-up, two of four (50%) experienced a significant increase in CD serological markers (>10× ULN) and/or the appearance of clinical symptoms. This led to diagnosis of classic or silent CD, while another two of four (50%) experienced a gradual decrease in anti-tTG and EMA antibodies serum titer. According to K. P. Newton and S. A. Singer, in a group of pediatric patients diagnosed with CD, 20% will be diagnosed with potential CD. Inside this group, 14% will experience a decrease in anti-tTG and EMA antibodies titer below the cut-off value during follow-up [12]. On the other hand, J. Bierła et al., who investigated patients with type 1 diabetes for CD, noticed an increased rate of CD within the study group. It expanded by 57% (21 + 12) over a three-year follow-up [11].

Furthermore, while following gluten-free diet, four of six (66%) patients with INS and CD experienced sustained remission or a reduction in the frequency of relapses. These results are difficult to compare with literature data because there are very few reports on this topic. T. Srivastava and colleagues observed patients with steroid-sensitive, frequently relapsing INS on a gluten-free diet for 6 months. A total of 29% of the study participants experienced no or fewer INS relapses after initiating the diet [5]. Another study by MJ Pérez-Sáez and colleagues evaluated a group of 16 patients with steroid-resistant INS on a gluten-free diet. After 4 weeks, 2 of 16 (12.5%) patients achieved remission of proteinuria. Proteinuria recurred after reintroduction of a gluten diet [4].

CD may be connected with IgA deficiency. The incidence of IgA deficiency in the study group and controls was comparable: 7% to 8%. CD was diagnosed in as many as 37.5% of patients with INS and IgA deficiency. The patient with CD in the control group had no IgA deficiency. IgA deficiency statistically occurs in approximately 0.17% of children in Europe. The prevalence of this primary immunodeficiency is probably higher, but due to its asymptomatic course, some patients remain undiagnosed [13]. The most common diseases associated with IgA deficiency are recurrent respiratory and gastrointestinal infections, allergies, and autoimmune diseases [14]. According to R. Yazdani and colleagues, the incidence of IgA deficiency in patients with CD is approximately 3% [13]. Among patients with IgA deficiency, CD occurs in 2–8% of patients [11]. The number of patients with INS, CD, and IgA deficiency in the study may have been different if the group of participants was larger.

CD has changed its clinical picture in recent decades, and the classic form is less frequent nowadays. According to the medical questionnaire in the study, patients with diagnosed CD had significantly higher incidence of skin rashes and mucus in stools during infancy compared to the non-celiac group. Abdominal pain, bloating, and failure to thrive in infancy were more common in patients with CD, but statistical significance was not confirmed. In the study, only two of nine patients (22%) reported periodic loose stools (types 5 and 6 on the Bristol scale). CD stigmata in patients with INS are difficult to search. First, growth retardation and osteoporosis are not only signs of CD but are also common complications of chronic steroid therapy. Furthermore, a reduction in vitamin D3 level may be a sign of CD but could also be due to hypoalbuminemia. Hypoalbuminemia and hypovolemia lead to an increase in red blood cell counts, making anemia (stigma of CD) harder to find. The medical literature reports CD to be diagnosed approximately a decade after first symptoms [11]. The classic form of CD is most often diagnosed in children under 2 years of age [11]. According to K. P. Newton and S. A. Singer, the ratio of symptomatic to asymptomatic patients in the pediatric population is 1:7 [12].

This study has limitations. As a single-center study, the retrospective research may be associated with some occurrence of bias. Limited participants in both subgroups and only a few participants with INS and CD may impact the outcomes. This issue needs further investigation.

## 6. Conclusions

To the best of our knowledge, the incidence of CD in INS is similar to other autoimmune diseases and exceeds the incidence rate in the general population. Moreover, CD can be asymptomatic or oligosymptomatic, so diagnostic tests should be performed in all patients with INS. A gluten-free diet in INS and CD patients may help to maintain complete remission or reduce the number of relapses. The results of the study require further investigation in a larger group of patients.

## Figures and Tables

**Table 1:** Celiac disease diagnostics in the study and control groups [Tissue transglutaminase IgA antibodies (tTG-IgA); Tissue transglutaminase IgG antibodies (tTG-IgG); Endomysial IgA antibodies (EMA-IgA); Endomysial IgG antibodies (EMA-IgG)].

	Idiopathic Nephrotic Syndrome	Control Group
Celiac disease	8%	2%
tTG IgA	8%	2%
tTG IgG	4.5%	2%
EMA IgA	5%	2%
EMA IgG	1.2%	2%
IgA deficiency	7%	8%
Gastroduodenoscopy	4%	0%
Skin biopsy	1%	0%
Presence of HLA DQ2DQ8	1%	0%

**Table 2:** Analysis of patients’ medical questionnaire.

	Patients with Celiac Disease	Patients Without Celiac Disease	*p*
	n = 9	n = 138	
	Prevalence		
Infancy			
Full-term delivery	1	0.95	1
Rash	0.77	0.34	0.026
Abdominal pain	0.55	0.22	0.068
Bloating	0.55	0.21	0.051
Colic	0.55	0.32	0.288
Constipation	0.33	0.17	0.486
Diarrhea	0.44	0.24	0.342
Mucus in stool	0.44	0.1	0.018
Blood in stool	0.11	0.03	0.757
Failure to thrive	0.37	0.1	0.098
Post-infancy period			
Atopic dermatitis	0.22	0.13	0.802
Allergic rhinitis	0.44	0.28	0.508
Allergic conjunctivitis	0.44	0.16	0.100
Bronchial asthma	0.11	0.07	1.0
Rash	0.33	0.29	1.0

**Table 3:** The impact of gluten-free diet on the course of idiopathic nephrotic syndrome.

Number	1	2	3	4	5	6	7	8
Age	8	9	10	11	12	13	17	18
Sex	M	F	M	M	F	F	F	F
Steroid-sensitive INS	Yes	Yes	Yes	Yes	No	Yes	Yes	Yes
Steroid-resistant INS	No	No	No	No	Yes	No	No	No
Frequently relapsing INS	No	Yes	No	No	-	No	No	No
Infrequently relapsing INS	Yes	No	Yes	Yes	-	Yes	Yes	Yes
Kidney biopsy	No	Yes	Yes	No	Yes	No	Yes	No
Genetic testing for INS	No	Yes	No	No	Yes	No	No	No
Second-line treatment	No	Yes	No	No	Yes	No	No	Yes
Gluten-free diet	No	Yes	Yes	Yes	Yes	Yes	Yes	No
Number of relapses before the diet	1	>10	3	4	1	2	3	8
Number of relapses after introducing the diet	-	>10	0	1	1	0	0	-
Follow-up period (years)	4	4	4	3	5	4	1	1

## Data Availability

The data analyzed in this study are available from the corresponding author upon reasonable request.

## Abbreviations

The following abbreviations are used in this manuscript:

INS	Idiopathic nephrotic syndrome
NS	Nephrotic syndrome
CD	Celiac disease
tTG	Transglutaminase
EMA	Endomysium
IgA	Immunoglobulin class A
IgG	Immunoglobulin class G
GCs	Glucocorticoids
ESPGHAN	European Society for Pediatric Gastroenterology, Hepatology, and Nutrition
